# The role of oestrogen in determining sexual dimorphism in energy balance

**DOI:** 10.1113/JP279501

**Published:** 2022-10-13

**Authors:** Anne Nicole De Jesus, Belinda A. Henry

**Affiliations:** ^1^ Metabolism, Obesity and Diabetes Program Biomedicine Discovery Institute Department of Physiology, Monash University Clayton Victoria Australia

**Keywords:** 17β‐oestradiol, energy expenditure, thermogenesis and food intake

## Abstract

Energy balance is determined by caloric intake and the rate at which energy is expended, with the latter comprising resting energy expenditure, physical activity and adaptive thermogenesis. The regulation of both energy intake and expenditure exhibits clear sexual dimorphism, with young women being relatively protected against weight gain and the development of cardiometabolic diseases. Preclinical studies have indicated that females are more sensitive to the satiety effects of leptin and insulin compared to males. Furthermore, females have greater thermogenic activity than males, whereas resting energy expenditure is generally higher in males than females. In addition to this, in post‐menopausal women, the decline in sex steroid concentration, particularly in oestrogen, is associated with a shift in the distribution of adipose tissue and overall increased propensity to gain weight. Oestrogens are known to regulate energy balance and weight homeostasis via effects on both food intake and energy expenditure. Indeed, 17β‐oestradiol treatment increases melanocortin signalling in the hypothalamus to cause satiety. Furthermore, oestrogenic action at the ventromedial hypothalamus has been linked with increased energy expenditure in female mice. We propose that oestrogen action on energy balance is multi‐faceted and is fundamental to determining sexual dimorphism in weight control. Furthermore, evidence suggests that the decline in oestrogen levels leads to increased risk of weight gain and development of cardiometabolic disease in women across the menopausal transition.

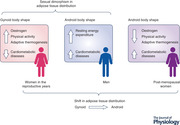

## Graphical abstract

**Abstract figure legend** A schematic illustration highlighting the key role of oestrogen in the protection against weight gain and cardiometabolic disease in young reproductive age women. High concentrations of oestrogens in young women are associated with differences in adipose tissue distribution, such that adipose is primarily accumulated in the gynoid subcutaneous regions compared to the android adipose phenotype typically seen in men and post‐menopausal women. In addition, circulating levels of oestrogen in women of reproductive age are associated with inherent changes in energy expenditure including increased physical activity and adaptive thermogenesis. These differences in body composition and energy balance are thought to underpin relative resistance to weight gain and improved metabolic health in young women.

## Introduction

Excess adiposity and obesity increase the risk of developing cardiometabolic diseases, which include hypertension, stroke, insulin resistance and type 2 diabetes (T2D) (Khan et al., 2018; Mokdad et al., 2003). Thus, overweight and obesity are associated with significantly increased risk of morbidity and mortality. In the simplest sense, obesity is caused by the imbalance in energy homeostasis whereby food intake exceeds energy expenditure; however, this does not account for the complex and multifactorial mechanisms that are essential to the control of body weight (Di Tecco et al., 2020; Salinero et al., 2018). Previous work has shown clear sexual dimorphism in the prevalence of obesity as well as the development of hypertension, glucose intolerance and type II diabetes. Women have evolved to typically have overall increased adiposity compared to men (Schorr et al., 2018). Despite this, pre‐menopausal women are considered to be relatively protected against weight gain and the development of cardiometabolic diseases compared to age‐matched men (Schorr et al., 2018). This protection against cardiovascular and metabolic disease is at least partly due to differences in body composition and the distribution of adipose tissue, where young women typically display adipose accumulation within the subcutaneous lower body regions (gynoid body shape) (Fried et al., 2015; Karastergiou et al., 2012). On the other hand, men display an android distribution of adipose tissue, which is characterised by accumulation within the visceral compartments (Fried et al., 2015; Karastergiou et al., 2012). This disparity in the distribution of adipose tissue is primarily driven by differing concentrations of sex steroids. Indeed, gender‐affirming hormone therapy can modulate the android: gynoid adipose ratio, where oestrogen therapy lowers the android: gynoid adipose ratio in transgender women and androgen therapy increases the android: gynoid ratio in transgender men (Bretherton et al., 2021). In addition, across the menopausal transition and with declining levels of ovarian steroids, women experience a shift in the distribution of adipose tissue from the gynoid to the android phenotype (Greendale et al., 2021) and this is clearly associated with increased risk of developing cardiometabolic diseases (Sari et al., 2019). It is important to highlight that not only is menopause associated with a change in body conformation, but women also experience generalised weight gain (Davis et al., 2012; Proietto, 2017), and it has been reported that overweight/obese women gain more weight in midlife than their lean counterparts (Proietto, 2017). However, to date, the mechanisms that underpin weight gain in women across mid‐life remain largely unknown.

Body weight and adiposity are primarily determined by caloric intake and the rate at which energy is expended. Energy expenditure has three major components: resting energy expenditure (REE)/basal metabolic rate (BMR), physical activity and adaptive thermogenesis. Numerous studies have shown sex differences in the regulation of both food intake and energy expenditure, with effects being both dependent and independent of sex steroid action. A good example of sexual dimorphism is that females are more responsive to the adipose‐derived hormone leptin and have enhanced melanocortin signalling in the brain, which may confer increased satiety (Clegg et al., 2006; Quarta et al., 2021; van Veen et al., 2020; Wang et al., 2018). To date, studies have also suggested that sex differences may result from inherent differences in developmental programming, X‐linked chromosomal genes, neuroanatomical differences of hypothalamic feeding circuitry as well as variations in sex steroids (Wang & Xu, 2019). In regard to the latter, there is strong evidence to suggest that oestrogens play a vital role in regulating body weight and metabolic function. There are three main oestrogenic hormones, oestriol, 17β‐oestradiol and oestrone, with 17β‐oestradiol being the predominant steroid synthesised in pre‐menopausal women (Cui et al., 2013). Indeed, studies have demonstrated that the beneficial metabolic effects of oestrogens are primarily via 17β‐oestradiol action. In particular, 17β‐oestradiol is known to exert reciprocal effects to reduce food intake and increase energy expenditure. This review will discuss the role of oestrogen in determining sexual dimorphism in the control of energy balance, with a particular emphasis on the regulation of energy expenditure.

## Sexual dimorphism and the role of oestrogen in determining weight gain: evidence from pre‐clinical models

Evidence shows that one's susceptibility to weight gain is determined by myriad genetic and physiological factors. Adult male mice have a greater propensity to gain weight, irrespective of diet, compared to their female counterparts (Hong et al., 2009). This protective effect against weight gain in females has primarily been attributed to circulating levels of oestrogen, since ovariectomy abolishes the sex differences seen in weight gain, with adult female mice gaining weight to a similar degree to males (Hong et al., 2009). Indeed, oestradiol benzoate treatment to ovariectomised female mice has been shown to prevent ovariectomy‐induced weight gain (Cavalcanti‐de‐Albuquerque et al., 2014). A further example of the importance of oestrogen in the control of body weight is the aromatase knockout (ArKO) murine model, whereby deletion of the enzyme responsible for converting androgens to oestrogens leads to obesity in male and female mice (Jones et al., 2000). It is also important to note that in general male mice are not only more susceptible to weight gain than female mice, but also exhibit an altered inflammatory profile as well as impaired insulin sensitivity, which increases the risk of developing secondary cardiometabolic complications including hypertension and T2D (Estrany et al., 2013). Thus, evidence suggests that murine models are similar to humans (as described above in Introduction) whereby circulating oestrogens exert numerous protective effects on metabolic health.

Oestrogenic actions are exerted via the nuclear oestrogen receptors α and β (ERα and ERβ) as well as the cell membrane G‐protein‐coupled oestrogen receptor (GPER). With regard to the classical nuclear receptors, studies in rodents indicate that the beneficial effects of oestrogen on body weight and metabolic function are primarily mediated via ERα (Roesch, 2006). Genetic deletion of ERα in both male and female mice leads to weight gain, insulin resistance and glucose intolerance (Heine et al., 2000). Furthermore, the central nervous system is integral to the protective metabolic effects of oestrogens, since brain‐specific deletion of ERα leads to obesity and increased adiposity in female mice, despite a compensatory increase in peripheral concentrations of 17β‐oestradiol (Xu et al., 2011). It is now widely recognised that 17β‐oestradiol acts at various sites within the central nervous system to reduce food intake and increase energy expenditure, which will be discussed in detail below.

In addition to the classical ERα, metabolic effects of oestrogen can also occur via GPER, previously referred to as GPR30 (Saito et al., 2015; Sharma & Prossnitz, 2021). In rodents, GPER is abundantly expressed in metabolically important tissues including adipose tissues, pancreatic islets and the hypothalamus (Davis et al., 2014; Hazell et al., 2009; Prossnitz et al., 2007; Shaklai et al., 2019). Indeed, a recent study demonstrated immunostaining of GPER within neurons, astrocytes and oligodendrocytes in the arcuate nucleus, paraventricular nucleus, lateral hypothalamus and ventromedial nucleus of the hypothalamus in male and female rats (Marraudino et al., 2021). Moreover, levels of immunostaining revealed sexual dimorphism whereby the total number of GPER‐immunostained cells was greater in the arcuate nucleus and the lateral hypothalamus of female than male rats (Marraudino et al., 2021). Despite this, genetic knockout studies have produced dichotomous findings with some studies showing that deletion of the GPER in mice can produce a similar metabolic phenotype to the ERα knockout mice with the onset of obesity and insulin resistance (Sharma & Prossnitz, 2021). Alternatively, studies have demonstrated that female GPER knockout mice have lowered body weights compared to males, which contrasts with the known effects of oestrogen to reduce weight gain (Wang et al., 2016). Thus, further work is required to delineate the physiological role of GPER in relaying the metabolic effects of 17β‐oestradiol.

## Neuroendocrine control of food intake

**Figure 1:**
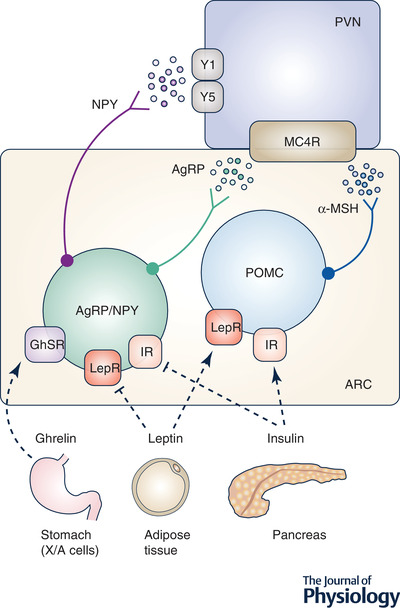
The hypothalamic subnuclei involved in the regulation of the food intake pathway The ARC houses the AgRP/NPY and POMC neurons. Leptin is released from adipose tissues and acts on the leptin receptors of the POMC neurons. On the other hand, insulin is released from the pancreas and acts on the insulin receptors of the POMC neurons. The activation of LepR and IR results in the inhibition of the AgRP/NPY neurons and thus decreased feeding. Ghrelin, released from the X/A‐like cells of the stomach, acts upon the GhSR located on the AgRP/NPY neurons, which results in the release of AgRP, acting as an endogenous antagonist against α‐MSH. NPY is also released, which acts on Y1/Y5 receptors located within the PVN, and alongside the effects of AgRP on MC4R, promote feeding. α‐MSH, α‐melanocyte stimulating hormone; AgRP, agouti‐related peptide; ARC, arcuate nucleus; E2, 17β‐oestradiol; ERα, oestrogen receptor α; GhSR, ghrelin receptors; IR, insulin receptors; LepR, leptin receptors; MC4R, melanocortin‐4 receptor; NPY, neuropeptide Y; POMC, pro‐opiomelanocortin; PVN, paraventricular nucleus; Y1, Y1 receptor; Y5, Y5 receptor.

Various endocrine factors including leptin, ghrelin, insulin and 17β‐oestradiol are integral to the control of food intake and energy expenditure (Briggs & Andrews, 2011; Cowley et al., 2001; Dodd et al., 2015; Gao et al., 2007; Wallen et al., 2001). The neural control of food intake involves the complex interplay between both reward and homeostatic pathways (Simon et al., 2017). The hypothalamus is known to be essential to the integration of information provided by peripheral signals to effect physiological control of energy balance. The satiety hormones, leptin and insulin, act at the proopiomelanocortin (POMC) and neuropeptide Y/agouti‐related protein (NPY/AgRP) neurons in the arcuate nucleus (Fig. 1). Both leptin and insulin depolarise POMC neurons leading to the release of the neurotransmitter α‐melanocyte‐stimulating hormone (α‐MSH), which reduces food intake via the melanocortin‐4 receptor (MC4R)‐expressing neurons located in the paraventricular nucleus (Cowley et al., 2001) (Fig. 1). In contrast, NPY/AgRP neurons in the arcuate nucleus are responsible for signalling hunger (Willesen et al., 1999) (Fig. 1). AgRP acts as an endogenous antagonist to inhibit α‐MSH binding at the MC4R and thus prevents the satiety signal from the POMC neurons (Pritchard et al., 2004) (Fig. 1). NPY increases food intake via the Y1 and Y5 receptors located on neurons within the paraventricular nucleus (Dube et al., 2000; Duhault et al., 2000; Pralong et al., 2002) (Fig. 1). Leptin and insulin directly activate POMC neurons and inhibit NPY/AgRP neurons, thus reducing food intake and causing satiety (Fig. 1). On the other hand, the hunger hormone, ghrelin, is secreted from X/A‐like cells of the stomach immediately prior to a meal (Stengel & Tache, 2012) and directly activates NPY/AgRP neurons (Fig. 1). It is important to note that factors that regulate food intake exert reciprocal effects to also regulate energy expenditure. For example, leptin acts at the brain to cause satiety but also increases energy expenditure, thus effecting weight loss in lean individuals (Henry et al., 2011, 2008; Heymsfield et al., 1999). Interestingly, pre‐clinical studies have demonstrated that 17β‐oestradiol acts in concert with leptin to regulate food intake, energy expenditure and body weight.

### Sexual dimorphism in the control of food intake: the interplay between leptin and oestrogen

There are clear sex differences in the neuroendocrine control of food intake. Various animal studies, in both rodents and sheep, have shown that the satiety effect of leptin is greater in females than males (Clarke et al., 2001; Clegg et al., 2006; Rosenbaum et al., 2001; Saad et al., 1997). Indeed, in rats, 17β‐oestradiol treatment to gonadectomised males increases leptin sensitivity so that the leptin‐induced decrease in food intake is similar to that seen in intact females (Clegg et al., 2006). Similarly, the effect of insulin and ghrelin on food intake is sexually dimorphic, with the satiety effect of insulin being greater in female than male rats, whereas the orexigenic effect of ghrelin is exacerbated in male compared to female rats (Clegg et al., 2006); importantly these effects have been attributed to differences in the circulating levels of 17β‐oestradiol. Thus, it has been suggested that a degree of cross‐talk exists between the mechanisms that underpin the satiety effects of oestrogens and classical metabolic hormones such as leptin and ghrelin. Despite this, a recent study demonstrated that oestradiol benzoate treatment of ovariectomised female mice had no effect on the ability of leptin to increase signal transducer and activator of transcription 3 (STAT3) phosphorylation (Kim et al., 2016). This indicates that oestrogen does not regulate body weight simply via enhancing leptin action. Instead, studies strongly suggest that 17β‐oestradiol directly regulates body weight via ERα‐expressing neurons within the hypothalamus. Oestrogen exerts numerous actions via ERα‐expressing neurons in the hypothalamus, including but not exclusive to, control of reproductive function and the hypothalamo–pituitary–gonadal axis, sexual behaviours, appetite and energy expenditure (Gieske et al., 2008; Martinez de Morentin et al., 2014; Santollo et al., 2007; Trouillet et al., 2022; van Veen et al., 2020). The latter two components will be discussed in detail below.

In both animals and humans, endogenous fluctuations in circulating 17β‐oestradiol levels have been linked to cyclical changes in food intake in females (Lyons et al., 1989). In women, food intake is lowest during the peri‐ovulatory phase of the menstrual cycle, when 17β‐oestradiol levels are highest (Kemnitz et al., 1989; Lyons et al., 1989). Oestrogenic effects to reduce food intake are primarily mediated via ERα, as the satiety effect of 17β‐oestradiol treatment is abolished in ERα knockout mice (Santollo et al., 2007). Further studies in mice suggest that the satiety effect of 17β‐oestradiol is primarily via the hypothalamic melanocortin pathway (Ainslie et al., 2001; Geary, 2001; Hirosawa et al., 2008; Roepke et al., 2010; Stincic et al., 2018). In female mice, altered expression of *Agrp* and *Npy* in the arcuate nucleus coincides with fluctuations in food intake across the ovarian cycle and genetically induced neurodegeneration of AgRP neurons abolishes cyclical changes in feeding (Olofsson et al., 2009). However, this effect appears to be indirect as, at least in mice, ERα is not co‐localised to the AgRP/NPY neuron (Olofsson et al., 2009). On the other hand, specific deletion of ERα in POMC neurons of the arcuate nucleus causes hyperphagia and obesity in female mice (Xu et al., 2011) and more recent studies suggest that specific subpopulations of POMC neurons may be integral to determining sex differences in energy balance (Hubbard et al., 2019; Quarta et al., 2021; Reilly et al., 2019; Wang et al., 2018).

Within the arcuate nucleus, POMC neurons are known to be heterogeneous, with studies suggesting that discrete topographical subpopulations may differentially regulate food intake, energy expenditure as well as peripheral glucose and lipid metabolism (Quarta et al., 2021). Female mice are known to have a greater number of POMC neurons than male mice (Wang et al., 2018) and genetic manipulation of POMC neuronal subpopulations leads to sexually dimorphic control of body weight in male and female mice (Wang et al., 2018). For example, deletion of the transcription factor Tap63 from POMC neurons elicits weight gain and obesity in female mice fed a high‐fat diet and thus abolishes the innate protection offered by oestrogen; there is little effect on weight gain in male mice (Wang et al., 2018). Interestingly, two studies have recently suggested that POMC neurons exert sexually divergent effects to preferentially regulate energy expenditure in females *versus* food intake in males. In mice, targeted mutation of the *Pomc* gene to abolish the synthesis of both α‐MSH and desacetyl‐α‐MSH causes hyperphagia in males, but in contrast, reduces energy expenditure in females (Hubbard et al., 2019). Similarly, the deletion of Gpr17 in POMC neurons causes significantly less weight gain in females due to enhanced locomotion and increased energy expenditure compared to males (Reilly et al., 2019). This highlights an important role for energy expenditure in determining the effects of sex steroids on energy homeostasis as well as the effects of sexual dimorphism in the control of body weight.

Although oestrogens are known to cause satiety, it is important to highlight that the effects of 17β‐oestradiol on food intake are frequently considered to be transient. In ovariectomised ewes, chronic 3 × 3 cm silastic implants containing 17β‐oestradiol causes an initial decrease in food intake; however, this returns to baseline within 5 days of treatment (Clarke et al., 2013). Furthermore, genetic deletion of the aromatase enzyme in male and female ArKO mice causes weight gain, which manifests in the absence of hyperphagia (Jones et al., 2000). To further highlight the possible role of energy expenditure in mediating oestrogen‐induced changes in body weight, Rogers et al. (2009) demonstrated that ovariectomy‐induced weight gain across 12 weeks was associated with reduced energy expenditure without an associated change in daily food intake (Rogers et al., 2009). This suggests that in the long‐term, oestrogens and in particular 17β‐oestradiol, acts to regulate body weight primarily via the modulation of energy expenditure.

## Mechanisms of energy expenditure

**Figure 2:**
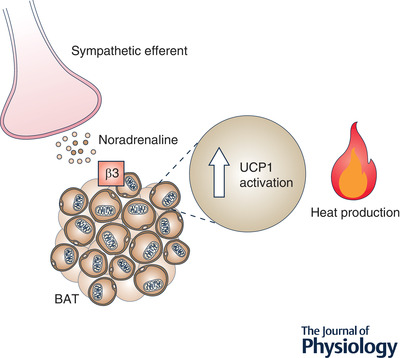
The activation of adaptive thermogenesis within brown adipose tissues The activation of the sympathetic nervous system results in the release of noradrenaline, which is a metabolic regulator of brown adipose tissues. A series of events activate UCP1, which generates heat and contributes to energy expenditure via adaptive thermogenesis. ARC, arcuate nucleus; BAT, brown adipose tissue; β3, β_3_‐adrenoceptor; UCP1, uncoupling protein‐1.

As indicated above, there are three major forms of energy expenditure, namely REE or BMR, physical activity, and adaptive thermogenesis. The latter refers to the dissipation of energy through specialised heat production and occurs in brown and beige adipocytes as well as skeletal muscle (Gong et al., 1997; Hilse et al., 2016; Walden et al., 2012). Thermogenesis in brown and beige adipocytes is regulated by both central and peripheral factors. Typically, thermogenic stimuli, including exposure to low ambient temperature or food consumption, are perceived by the brain leading to activation of the sympathetic nervous system. In turn, noradrenaline via action at β‐adrenergic receptors on brown adipocytes causes activation of uncoupling protein (UCP)‐1, which creates a proton leak across the inner mitochondrial membrane; this process of uncoupled respiration leads to the dissipation of energy via heat production (Fig. 2). Exogenous factors including peroxisome proliferator‐activated receptor γ (PPARγ) agonists and β_3_‐adrenergic agonists can increase thermogenesis via activation of brown adipocytes or recruitment of beige adipocytes, which occurs through the process known as ‘browning’ (Petrovic et al., 2010). In addition to adipose (brown or beige) thermogenesis, myocytes are capable of producing heat through similar futile cellular pathways involving both mitochondrial uncoupling through UCP3 and futile calcium cycling (Bal & Periasamy, 2020; Clarke et al., 2012).

To date, various studies have indicated that the physiological control of energy expenditure is sexually dimorphic; however, the direction of this effect is dependent on the specific component of energy expenditure. For example, REE is greater in men than women, but on the other hand, women exhibit increased thermogenesis compared to men (Arciero et al., 1993; Au‐Yong et al., 2009; Cypess et al., 2009; Ferraro et al., 1992; Fletcher et al., 2020; Kirkby et al., 2004; Ouellet et al., 2011). Furthermore, pre‐clinical data from murine models clearly demonstrate that oestrogens act at the ventromedial hypothalamus (VMH) to regulate multiple facets of energy expenditure including both physical activity and adaptive thermogenesis (Krause et al., 2021; Martinez de Morentin et al., 2014; van Veen et al., 2020).

### Sexually dimorphic control and the impact of menstrual cyclicity on resting energy expenditure

REE is defined as the energy expended to maintain physiological functions essential to life, and this contributes to approximately 60−70% of total daily energy expenditure (Blundell et al., 2012). The primary determinant of REE is lean body mass, and as such, men typically have a higher rate of REE than women (Arciero et al., 1993; Rodriguez et al., 2002; Souba, 1997). In addition to this frank sexual dimorphism, a recent systematic review provided evidence to suggest that there is a small effect of menstrual cyclicity on REE in pre‐menopausal women (Benton et al., 2020). In this case, REE may be slightly increased in women during the luteal phase compared with the follicular phase of the menstrual cycle (Benton et al., 2020). Whether this coincides with fluctuations in sex steroids remains unknown and requires further investigation. Additionally, a reduction in REE has been reported across the menopausal transition, and REE is thought to be lower in post‐menopausal women due to a decline in lean mass (Van Pelt et al., 1997). Despite this, in young women, suppression of sex steroid levels by chronic administration of a gonadotropin‐releasing hormone (GnRH) agonist (for 5 months) decreased REE; this effect was attenuated by 17β‐oestradiol replacement (Melanson et al., 2015). This suggests that oestrogen may exert some effect to regulate energy expenditure via modulating metabolic rate, at least in young women of reproductive age.

### Physical activity is regulated via oestrogen in a sexually dimorphic manner

**Figure 3:**
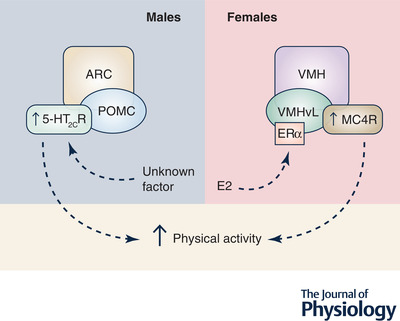
The hypothalamic subnuclei involved in the regulation of physical activity in males and females It is proposed that a subpopulation within the VMH, known as the VMHvl, co‐expresses both ERα and MC4R. 17β‐Oestradiol activates the VMHvl, which leads to the upregulation of the MC4R, thereby promoting physical activity in females. In males, POMC within the ARC promotes physical activity via the activation of the 5‐HT_2C_R, which is a serotonin receptor, regulated by an unknown factor. 5‐HT_2C_R, 5‐hydroxytryptamine 2c receptor; ARC, arcuate nucleus; E2, 17β‐oestradiol; ERα, oestrogen receptor α; MC4R, melanocortin‐4 receptor; POMC, pro‐opiomelanocortin; VMH, ventromedial hypothalamus; VMHvl, ventrolateral ventromedial hypothalamus.

Physical activity comprises both spontaneous activity or non‐exercise activity thermogenesis and volitional exercise. Various endogenous and exogenous factors are known to influence levels of physical activity including genetic variance, biological determinants (including exercise ability and motivation), as well as social‐environmental factors such as diet (Lightfoot et al., 2018). Indeed, sex steroids are considered to be a biological factor that can influence physical activity (Lightfoot et al., 2018). Studies conducted using rodent models show that ovariectomy causes a marked decrease in spontaneous physical activity, which can be reversed by 17β‐oestradiol treatment (Gorzek et al., 2007; Izumo et al., 2012; Krause et al., 2021). In addition, longitudinal studies have demonstrated that post‐menopausal women are less likely to engage in physical activity than the same individuals prior to the menopausal transition (Poehlman, 2002). To further elucidate the role of oestrogen in regulating physical activity in women, Melanson et al. (2015) chronically reduced ovarian steroid levels via administration of a GnRH agonist, which led to a reduction in exercise‐associated energy expenditure; this effect was prevented by 17β‐oestradiol treatment. Together these data indicate that oestrogen may exert effects, at least in women, to enhance physical activity. To further support this, work in mice indicates that oestradiol acts at the VMH to regulate energy expenditure, via increased physical activity and adaptive thermogenesis (Koch, 1990; Krause et al., 2021; van Veen et al., 2020) (Fig. 3). Chemogenic activation of ERα‐expressing neurons in the VMH leads to an increase in spontaneous activity in both male and female mice (van Veen et al., 2020). However, the VMH is known to be a sexually dimorphic nucleus, and consistent with this, there are discrete subpopulations of neurons that are differentially involved in the regulation of physical activity in males and females (Krause et al., 2021; van Veen et al., 2020) (Fig. 3). In female mice, ERα is abundantly expressed in the ventrolateral VMH, with lower expression in males (Koch, 1990). Furthermore, oestradiol benzoate action within this subnucleus up‐regulates MC4R levels and chemogenetic stimulation of the ventrolateral VMH neurons increases locomotor activity causing weight loss in ovariectomised females (Krause et al., 2021) (Fig. 3). In contrast, the serotonin 5‐HT_2C_ receptors that are expressed on POMC neurons of the arcuate nucleus appear to be important functional regulators of physical activity in male but not female mice (Burke et al., 2016) (Fig. 3). Thus, the neural control of physical activity is clearly sexually dimorphic, and in females oestrogenic action to enhance physical activity appears to primarily occur within MC4R‐expressing neurons of the ventrolateral VMH.

### Sexual dimorphism in adaptive thermogenesis

In humans, the distribution and prevalence of brown adipose tissue (BAT) changes across life. BAT is most abundant in newborn infants where it is detected within the interscapular, peri‐renal and neck regions (Lean et al., 1986; Lidell, 2019). During early life, BAT is essential to the maintenance of core body temperature; however, levels rapidly decline once skeletal muscle attains the ability to produce heat via shivering (Cannon & Nedergaard, 2004). Historically, dogma stipulated that unlike in rodents, in humans BAT rapidly declines during the neonatal period to reach negligible levels in adulthood (Cannon & Nedergaard, 2004). However, more recent work demonstrates that at the time of puberty there is a reinstatement of BAT, but the BAT is redistributed and is primarily located within the neck, supraclavicular and paravertebral regions (Ikeda et al., 2018; Zingaretti et al., 2009). It has been estimated that adult humans have approximately 50 g of BAT, and this can account for up to 10% of total energy expenditure (Rothwell & Stock, 1983). Moreover, positron‐emission tomography–computed tomography (PET‐CT) studies measuring ^18^F‐fluoro‐2‐deoxy‐d‐glucose uptake show that BAT is most abundant in lean humans, and is reduced in individuals with obesity and T2D (Saito et al., 2009) and with ageing (Saito et al., 2009). Interestingly, a number of retrospective PET‐CT studies have indicated that women have greater levels of BAT than men, which aligns with their overall greater levels of adiposity (Au‐Yong et al., 2009; Cypess et al., 2009; Fletcher et al., 2020; Ouellet et al., 2011). Furthermore, recent studies have demonstrated that infra‐red thermography can be used to quantify changes in BAT activity via measurement of longitudinal changes in supraclavicular skin temperature (Fuller‐Jackson et al., 2020). Studies have used thermography to demonstrate that the change in supraclavicular temperature in response to thermogenic stimuli, including cold exposure and meal intake, is greater in women than men (Fuller‐Jackson et al., 2020). However, it is important to note that recent work has produced conflicting data suggesting that women have a lower supraclavicular BAT volume, but increased levels of BAT in the dorsocervical region (Fletcher et al., 2020). Therefore, further work is required to fully understand the sexually dimorphic nature of BAT in humans. Nonetheless, there is emerging evidence from rodent models that clearly identifies several mechanisms that may underpin differences in BAT activity between males and females.

Under *ad libitum* feeding conditions, female rats have a greater capacity to recruit BAT than their male counterparts (Valle et al., 2007). Earlier studies have outlined several factors that contribute to such differences including sex‐related dimorphism in BAT morphology. For example, BAT mitochondria in females are dense and are composed of longer cristae than those in males (Justo et al., 2005; Rodriguez‐Cuenca et al., 2002). Furthermore, the lipid profile of BAT is sexually dimorphic whereby brown adipocytes exhibit a distinct fatty acid profile, with arachidonic and stearic acids being predominant in females whereas elevated triglyceride content is evident in males (Hoene et al., 2014); this may render BAT more active in female than male rats. In addition to these morphological differences, sex steroids, in particular oestrogen, are known to alter BAT function. Indeed, the enhanced response of BAT to cold and dietary stimuli in women is correlated with circulating concentrations of 17β‐oestradiol (Fuller‐Jackson et al., 2020).

### Role of oestrogen in the control of adaptive thermogenesis

In ovariectomised rats, 17β‐oestradiol treatment causes profound weight loss compared to pair‐fed controls, providing direct evidence to indicate that 17β‐oestradiol controls body weight via regulation of appetite and non‐appetite pathways (Martinez de Morentin et al., 2014). Indeed, the aforementioned study demonstrates that 17β‐oestradiol treatment increases the expression of key thermogenic markers including UCP1, PPARγ coactivator 1α (PGC‐1α) and PPARγ in BAT (Martinez de Morentin et al., 2014), which is indicative that BAT plays a critical role in 17β‐oestradiol control of bodyweight.

**Figure 4:**
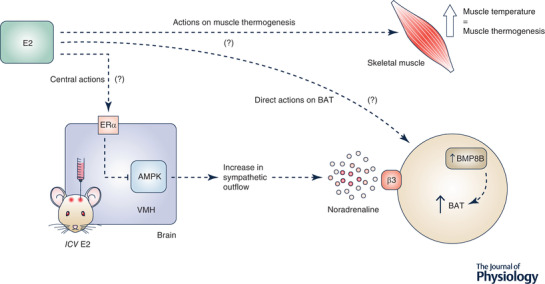
The proposed mechanisms involved in thermogenic activation via 17β‐oestradiol It is proposed that 17β‐oestradiol acts via three target sites to induce thermogenesis. Mechanism 1 involves central actions within the VMH whereby 17β‐oestradiol acts on ERα, which leads to the inhibition of AMPK. As a result, there is a subsequent increase in sympathetic outflow, and thus the release of noradrenaline. Mechanism 2 involves direct actions of 17β‐oestradiol on BAT, which leads to the activation of endocrine factors such as BMP8B, and thus results in BAT thermogenesis. It is also speculated that 17β‐oestradiol contributes to thermogenesis by acting on skeletal muscles to induce muscle thermogenesis. AMPK, AMP‐activated protein kinase; BAT, brown adipose tissue; BMP8B, bone morphometric protein 8b; E2, 17β‐oestradiol; ERα, oestrogen receptor α; VMH, ventromedial hypothalamus.

Although earlier work indicated that oestrogens may act directly at brown adipocytes to regulate thermogenesis via increased expression of bone morphogenic protein 8b (Grefhorst et al., 2015) (Fig. 4), more recent studies in rodents provide strong evidence that oestrogens increase BAT thermogenesis via action at the brain to modulate sympathetic outflow. In mice, specific deletion of ERα in the VMH via either delivery of adeno‐associated inhibitory viral vectors (Musatov et al., 2007) or targeted genetic deletion of ERα in SF1‐expressing neurons (Xu et al., 2011) causes obesity. In both circumstances, weight gain is not associated with any change in food intake, but coincides with impaired BAT thermogenesis (Martinez de Morentin et al., 2014). Indeed, at least in murine models, 17β‐oestradiol inhibits the phosphorylation of AMP‐activated protein kinase (AMPK) in neurons in the VMH, which in turn leads to activation of the sympathetic nervous system and the induction of BAT thermogenesis (Martinez de Morentin et al., 2014; Xu et al., 2011) (Fig. 4). Thus, in mice, the VMH is a central hub that is integral to relaying the effect of oestradiol on energy expenditure, where discrete subpopulations of neurons are important for relaying the effects on either physical activity (as described above) or BAT thermogenesis. It is important to note that the effects of oestrogen on adaptive thermogenesis, however, are not restricted to BAT as oestradiol also increases thermogenesis in beige adipocytes as well as skeletal muscle. In rodents, previous work has shown sexual dimorphism in the browning of white adipose tissue depots, where females exhibit greater ‘browning’ than males (Kim et al., 2016). In this regard, treatment with the β_3_‐adrenoceptor agonist CL316243 increased the expression of brown‐like adipocyte markers including UCP1 in both the gonadal and inguinal adipose tissue depots of female mice, but this effect was restricted to the inguinal fat of male mice (Kim et al., 2016). Furthermore, ovarian failure and therefore oestrogen depletion induced by 4‐vinylcyclohexene diepoxide treatment impaired beige cell recruitment in gonadal adipose tissue of female mice (Kim et al., 2016). Similarly, oestradiol benzoate treatment of ovariectomised ewes increases heat production in both retroperitoneal adipose tissue and skeletal muscle (Clarke et al., 2013), indicative of enhanced adipose and muscle thermogenesis, respectively. Furthermore, repeated injection of 17β‐oestradiol causes a sustained increase in muscle temperature in ovariectomised ewes (Clarke et al., 2013), which further supports a role for oestrogens in increasing thermogenesis across multiple tissue sites, including brown and beige adipocytes as well as skeletal muscle. Thus, oestrogen regulation of adaptive thermogenesis appears to be an important physiological component that determines sexual dimorphism in the control of energy homeostasis and metabolic function.

### Kisspeptin: a novel mediator of oestrogen induced changes in energy expenditure

Recent work has suggested that kisspeptin is a novel regulator of body weight via modulation of energy expenditure (Tolson et al., 2014; Velasco et al., 2019). Given that kisspeptin is an integral mediator of oestrogen feedback at the level of the hypothalamus, relaying both positive and negative feedback to the hypothalamo–pituitary–gonadal axis (Franceschini et al., 2006; Gottsch et al., 2004; Smith et al., 2005), it remains possible that the kisspeptin neurons may also be a conduit transmitting the effects of 17β‐oestradiol on energy expenditure (de Roux et al., 2003; Gottsch et al., 2004). In sheep, hypothalamic Kiss1‐containing neurons co‐express the leptin receptor, and furthermore reciprocal connections exist between the kisspeptin and the appetite‐regulating NPY and POMC neurons of the arcuate nucleus (Backholer et al., 2010). The kisspeptin receptor (KISS1R) is widely expressed within the hypothalamic brain regions, specifically within the arcuate nucleus. In female mice, genetic deletion of *Kiss1r* leads to weight gain and increased adiposity, which manifests in a reduction in energy expenditure (Tolson et al., 2016, 2014). The decrease in energy expenditure is multifactorial, where *Kiss1r* knockout mice exhibit lower locomotion, oxygen consumption and BAT thermogenesis (Tolson et al., 2016, 2020). Interestingly, this phenotype appears to be sexually dimorphic with a greater effect in females than males (Tolson et al., 2016, 2014). Furthermore, in female mice, ovariectomy control studies illustrate that weight gain is likely to occur via both oestrogen‐dependent and ‐independent mechanisms, where the latter is directly due to disruption in kisspeptin signalling (Tolson et al., 2014). This was further investigated in the study of Velasco et al. (2019), where *Kiss1r* expression was reinstated in the GnRH neurons of global *Kiss1r* knockout mice, which restored reproductive function and therefore sex steroid concentrations (Velasco et al., 2019). In comparison to the global *Kiss1r* knockout group, reinstatement of KISS1R in GnRH neurons obviated the increase in body weight and fat mass; however, the authors did not measure any indices of energy expenditure. Thus, further work is required to delineate the role of sex steroids in mediating changes in energy expenditure in *Kiss1r* knockout mice and the degree in which kisspeptin is involved in regulating energy expenditure, independent of sex steroid action.

## Conclusions

The control of body weight and energy homeostasis is sexually dimorphic. In rodents, females have greater satiety than males, which manifests via increased innate activity of POMC neurons. Indeed, 17β‐oestradiol binds to ERα colocalised to POMC neurons to reduce food intake. In addition to this, females have increased BAT volumes, leading to enhanced thermogenic activity. Indeed, in women, increased circulating levels of 17β‐oestradiol are associated with enhanced ability to activate BAT in response to thermogenic stimuli. The effect of 17β‐oestradiol on energy expenditure, however, is not solely attributable to changes in thermogenesis. In mice, 17β‐oestradiol acts at MC4R expressing neurons within the VMH to increase physical activity, and in women treatment with a GnRH agonist decreases 17β‐oestradiol levels causing a decline in both resting energy expenditure and physical activity. Thus, the effects of oestrogen to promote satiety and to increase overall energy expenditure are considered as key mechanisms underpinning sexual dimorphism in the control of body weight. Indeed, in animal models and in humans, females of reproductive age are less susceptible to weight gain than males, and across the menopausal transition women not only experience a change in body composition with altered adipose tissue distribution but also generalised weight gain. Thus, further understanding of the physiological mechanisms that underpin sexual dimorphism and sex steroid regulation of body weight has important ramifications for preventing weight gain in post‐menopausal women. Steroidal treatments such as hormone replacement therapy, gender affirming hormonal therapy and hormonal contraceptives are widely prescribed across various stages of life. Moreover, hormonal therapies are typically prescribed across many years, which may also lead to detrimental cardiometabolic risk in itself (Diaz et al., 2012; Rossouw et al., 2002; Todd et al., 1999). Despite their wide usage, very little is known as to how these exogenous steroids impact on metabolic function and energy balance. It is therefore imperative to understand how clinically used exogenous sex steroids alter energy‐balance pathways to impact on weight regulation and the altered risk of developing metabolic disease. In addition, understanding sexual dimorphism will be important to the design of new‐generation metabolic and weight loss pharmacotherapies that can be tailored to males and females, and therefore improve treatment strategies for all sexes.

## Additional information

### Competing interests

The authors (Anne Nicole De Jesus and Belinda A. Henry) have no conflict or competing interests to declare.

### Author contributions

Anne Nicole De Jesus contributed to the preparation and editing of the manuscript. She was responsible for producing each of the Figures contained within the manuscript. Belinda A. Henry contributed to the writing and final editing of the manuscript. Both authors have read and approved the final version of this manuscript and agree to be accountable for all aspects of the work in ensuring that questions related to the accuracy or integrity of any part of the work are appropriately investigated and resolved. All persons designated as authors qualify for authorship, and all those who qualify for authorship are listed.

### Funding

This work was supported by an Advancing Women's Research Success Grant, Monash University awarded to B.A.H.

## Supporting information




Peer Review History

## References

[tjp15279-bib-0001] Ainslie, D. A. , Morris, M. J. , Wittert, G. , Turnbull, H. , Proietto, J. , & Thorburn, A. W. (2001). Estrogen deficiency causes central leptin insensitivity and increased hypothalamic neuropeptide Y. International Journal of Obesity and Related Metabolic Disorders, 25(11), 1680–1688. 11753591 10.1038/sj.ijo.0801806

[tjp15279-bib-0002] Arciero, P. J. , Goran, M. I. , & Poehlman, E. T. (1993). Resting metabolic rate is lower in women than in men. Journal of Applied Physiology, 75(6), 2514–2520. 8125870 10.1152/jappl.1993.75.6.2514

[tjp15279-bib-0003] Au‐Yong, I. T. , Thorn, N. , Ganatra, R. , Perkins, A. C. , & Symonds, M. E. (2009). Brown adipose tissue and seasonal variation in humans. Diabetes, 58(11), 2583–2587. 19696186 10.2337/db09-0833 PMC2768171

[tjp15279-bib-0004] Backholer, K. , Smith, J. T. , Rao, A. , Pereira, A. , Iqbal, J. , Ogawa, S. , Li, Q. , & Clarke, I. J. (2010). Kisspeptin cells in the ewe brain respond to leptin and communicate with neuropeptide Y and proopiomelanocortin cells. Endocrinology, 151(5), 2233–2243. 20207832 10.1210/en.2009-1190

[tjp15279-bib-0005] Bal, N. C. , & Periasamy, M. (2020). Uncoupling of sarcoendoplasmic reticulum calcium ATPase pump activity by sarcolipin as the basis for muscle non‐shivering thermogenesis. Philosophical Transactions of the Royal Society of London Series B: Biological Sciences, 375(1793), 20190135. 31928193 10.1098/rstb.2019.0135 PMC7017432

[tjp15279-bib-0006] Benton, M. J. , Hutchins, A. M. , & Dawes, J. J. (2020). Effect of menstrual cycle on resting metabolism: A systematic review and meta‐analysis. PLoS One, 15(7), e0236025. 32658929 10.1371/journal.pone.0236025 PMC7357764

[tjp15279-bib-0007] Blundell, J. E. , Caudwell, P. , Gibbons, C. , Hopkins, M. , Naslund, E. , King, N. , & Finlayson, G. (2012). Role of resting metabolic rate and energy expenditure in hunger and appetite control: A new formulation. Disease Models & Mechanisms, 5(5), 608–613. 22915022 10.1242/dmm.009837 PMC3424457

[tjp15279-bib-0008] Bretherton, I. , Spanos, C. , Leemaqz, S. Y. , Premaratne, G. , Grossmann, M. , Zajac, J. D. , & Cheung, A. S. (2021). Insulin resistance in transgender individuals correlates with android fat mass. Therapeutic Advances in Endocrinology and Metabolism, 12, 204201882098568. 10.1177/2042018820985681 PMC7841663 33552464

[tjp15279-bib-0009] Briggs, D. I. , & Andrews, Z. B. (2011). Metabolic status regulates ghrelin function on energy homeostasis. Neuroendocrinology, 93(1), 48–57. 21124019 10.1159/000322589

[tjp15279-bib-0010] Burke, L. K. , Doslikova, B. , D'Agostino, G. , Greenwald‐Yarnell, M. , Georgescu, T. , Chianese, R. , Martinez de Morentin, P. B. , Ogunnowo‐Bada, E. , Cansell, C. , Valencia‐Torres, L. , Garfield, A. S. , Apergis‐Schoute, J. , Lam, D. D. , Speakman, J. R. , Rubinstein, M. , Low, M. J. , Rochford, J. J. , Myers, M. G. , … Heisler, L. K. (2016). Sex difference in physical activity, energy expenditure and obesity driven by a subpopulation of hypothalamic POMC neurons. Molecular Metabolism, 5(3), 245–252. 26977396 10.1016/j.molmet.2016.01.005 PMC4770275

[tjp15279-bib-0011] Cannon, B. , & Nedergaard, J. (2004). Brown adipose tissue: Function and physiological significance. Physiological Reviews, 84(1), 277–359. 14715917 10.1152/physrev.00015.2003

[tjp15279-bib-0012] Cavalcanti‐de‐Albuquerque, J. P. , Salvador, I. C. , Martins, E. L. , Jardim‐Messeder, D. , Werneck‐de‐Castro, J. P. , Galina, A. , & Carvalho, D. P. (2014). Role of estrogen on skeletal muscle mitochondrial function in ovariectomized rats: A time course study in different fiber types. Journal of Applied Physiology, 116(7), 779–789. 24458744 10.1152/japplphysiol.00121.2013

[tjp15279-bib-0013] Clarke, I. J. , Tilbrook, A. J. , Turner, A. I. , Doughton, B. W. , & Goding, J. W. (2001). Sex, fat and the tilt of the earth: Effects of sex and season on the feeding response to centrally administered leptin in sheep. Endocrinology, 142(6), 2725–2728. 11356725 10.1210/endo.142.6.8318

[tjp15279-bib-0014] Clarke, S. D. , Clarke, I. J. , Rao, A. , Cowley, M. A. , & Henry, B. A. (2012). Sex differences in the metabolic effects of testosterone in sheep. Endocrinology, 153(1), 123–131. 22128020 10.1210/en.2011-1634

[tjp15279-bib-0015] Clarke, S. D. , Clarke, I. J. , Rao, A. , Evans, R. G. , & Henry, B. A. (2013). Differential effects of acute and chronic estrogen treatment on thermogenic and metabolic pathways in ovariectomized sheep. Endocrinology, 154(1), 184–192. 23183185 10.1210/en.2012-1758

[tjp15279-bib-0016] Clegg, D. J. , Brown, L. M. , Woods, S. C. , & Benoit, S. C. (2006). Gonadal hormones determine sensitivity to central leptin and insulin. Diabetes, 55(4), 978–987. 16567519 10.2337/diabetes.55.04.06.db05-1339

[tjp15279-bib-0017] Cowley, M. A. , Smart, J. L. , Rubinstein, M. , Cerdan, M. G. , Diano, S. , Horvath, T. L. , Cone, R. D. , & Low, M. J. (2001). Leptin activates anorexigenic POMC neurons through a neural network in the arcuate nucleus. Nature, 411(6836), 480–484. 11373681 10.1038/35078085

[tjp15279-bib-0018] Cui, J. , Shen, Y. , & Li, R. (2013). Estrogen synthesis and signaling pathways during aging: from periphery to brain. Trends in Molecular Medicine, 19(3), 197–209. 23348042 10.1016/j.molmed.2012.12.007 PMC3595330

[tjp15279-bib-0019] Cypess, A. M. , Lehman, S. , Williams, G. , Tal, I. , Rodman, D. , Goldfine, A. B. , Kuo, F. C. , Palmer, E. L. , Tseng, Y. H. , Doria, A. , Kolodny, G. M. , & Kahn, C. R. (2009). Identification and importance of brown adipose tissue in adult humans. New England Journal of Medicine, 360(15), 1509–1517. 19357406 10.1056/NEJMoa0810780 PMC2859951

[tjp15279-bib-0020] Davis, K. E. , Carstens, E. J. , Irani, B. G. , Gent, L. M. , Hahner, L. M. , & Clegg, D. J. (2014). Sexually dimorphic role of G protein‐coupled estrogen receptor (GPER) in modulating energy homeostasis. Hormones and Behavior, 66(1), 196–207. 24560890 10.1016/j.yhbeh.2014.02.004 PMC4051842

[tjp15279-bib-0021] Davis, S. R. , Castelo‐Branco, C. , Chedraui, P. , Lumsden, M. A. , Nappi, R. E. , Shah, D. , & Villaseca, P. , Writing Group of the International Menopause Society for World Menopause D (2012). Understanding weight gain at menopause. Climacteric, 15(5), 419–429. 22978257 10.3109/13697137.2012.707385

[tjp15279-bib-0022] de Roux, N. , Genin, E. , Carel, J. C. , Matsuda, F. , Chaussain, J. L. , & Milgrom, E. (2003). Hypogonadotropic hypogonadism due to loss of function of the KiSS1‐derived peptide receptor GPR54. Proceedings of the National Academy of Sciences, USA, 100(19), 10972–10976. 10.1073/pnas.1834399100 PMC196911 12944565

[tjp15279-bib-0023] Di Tecco, C. , Fontana, L. , Adamo, G. , Petyx, M. , & Iavicoli, S. (2020). Gender differences and occupational factors for the risk of obesity in the Italian working population. BMC Public Health, 20(1), 706. 32416721 10.1186/s12889-020-08817-z PMC7229582

[tjp15279-bib-0024] Diaz, M. , Chacon, M. R. , Lopez‐Bermejo, A. , Maymo‐Masip, E. , Salvador, C. , Vendrell, J. , de Zegher, F. , & Ibanez, L. (2012). Ethinyl estradiol‐cyproterone acetate versus low‐dose pioglitazone‐flutamide‐metformin for adolescent girls with androgen excess: Divergent effects on CD163, TWEAK receptor, ANGPTL4, and LEPTIN expression in subcutaneous adipose tissue. Journal of Clinical Endocrinology and Metabolism, 97(10), 3630–3638. 22791755 10.1210/jc.2012-1754

[tjp15279-bib-0025] Dodd, G. T. , Decherf, S. , Loh, K. , Simonds, S. E. , Wiede, F. , Balland, E. , Merry, T. L. , Munzberg, H. , Zhang, Z. Y. , Kahn, B. B. , Neel, B. G. , Bence, K. K. , Andrews, Z. B. , Cowley, M. A. , & Tiganis, T. (2015). Leptin and insulin act on POMC neurons to promote the browning of white fat. Cell, 160(1–2), 88–104. 25594176 10.1016/j.cell.2014.12.022 PMC4453004

[tjp15279-bib-0026] Dube, M. G. , Horvath, T. L. , Kalra, P. S. , & Kalra, S. P. (2000). Evidence of NPY Y5 receptor involvement in food intake elicited by orexin A in sated rats. Peptides, 21(10), 1557–1560. 11068104 10.1016/s0196-9781(00)00311-9

[tjp15279-bib-0027] Duhault, J. , Boulanger, M. , Chamorro, S. , Boutin, J. A. , Della Zuana, O. , Douillet, E. , Fauchere, J. L. , Feletou, M. , Germain, M. , Husson, B. , Vega, A. M. , Renard, P. , & Tisserand, F. (2000). Food intake regulation in rodents: Y5 or Y1 NPY receptors or both? Canadian Journal of Physiology and Pharmacology, 78(2), 173–185. 10737680

[tjp15279-bib-0028] Estrany, M. E. , Proenza, A. M. , Gianotti, M. , & Llado, I. (2013). High‐fat diet feeding induces sex‐dependent changes in inflammatory and insulin sensitivity profiles of rat adipose tissue. Cell Biochemistry and Function, 31(6), 504–510. 23112138 10.1002/cbf.2927

[tjp15279-bib-0029] Ferraro, R. , Lillioja, S. , Fontvieille, A. M. , Rising, R. , Bogardus, C. , & Ravussin, E. (1992). Lower sedentary metabolic rate in women compared with men. Journal of Clinical Investigation, 90(3), 780–784. 1522233 10.1172/JCI115951 PMC329930

[tjp15279-bib-0030] Fletcher, L. A. , Kim, K. , Leitner, B. P. , Cassimatis, T. M. , O'Mara, A. E. , Johnson, J. W. , Halprin, M. S. , McGehee, S. M. , Brychta, R. J. , Cypess, A. M. , & Chen, K. Y. (2020). Sexual dimorphisms in adult human brown adipose tissue. Obesity, 28(2), 241–246. 31970907 10.1002/oby.22698 PMC6986330

[tjp15279-bib-0031] Franceschini, I. , Lomet, D. , Cateau, M. , Delsol, G. , Tillet, Y. , & Caraty, A. (2006). Kisspeptin immunoreactive cells of the ovine preoptic area and arcuate nucleus co‐express estrogen receptor alpha. Neuroscience Letters, 401(3), 225–230. 16621281 10.1016/j.neulet.2006.03.039

[tjp15279-bib-0032] Fried, S. K. , Lee, M. J. , & Karastergiou, K. (2015). Shaping fat distribution: New insights into the molecular determinants of depot‐ and sex‐dependent adipose biology. Obesity, 23(7), 1345–1352. 26054752 10.1002/oby.21133 PMC4687449

[tjp15279-bib-0033] Fuller‐Jackson, J. P. , Dordevic, A. L. , Clarke, I. J. , & Henry, B. A. (2020). Effect of sex and sex steroids on brown adipose tissue heat production in humans. European Journal of Endocrinology, 183(3), 343–355. 32508310 10.1530/EJE-20-0184

[tjp15279-bib-0034] Gao, Q. , Mezei, G. , Nie, Y. , Rao, Y. , Choi, C. S. , Bechmann, I. , Leranth, C. , Toran‐Allerand, D. , Priest, C. A. , Roberts, J. L. , Gao, X. B. , Mobbs, C. , Shulman, G. I. , Diano, S. , & Horvath, T. L. (2007). Anorectic estrogen mimics leptin's effect on the rewiring of melanocortin cells and Stat3 signaling in obese animals. Nature Medicine, 13(1), 89–94. 10.1038/nm1525 17195839

[tjp15279-bib-0035] Geary, N. (2001). Estradiol, CCK and satiation. Peptides, 22(8), 1251–1263. 11457518 10.1016/s0196-9781(01)00449-1

[tjp15279-bib-0036] Gieske, M. C. , Kim, H. J. , Legan, S. J. , Koo, Y. , Krust, A. , Chambon, P. , & Ko, C. (2008). Pituitary gonadotroph estrogen receptor‐alpha is necessary for fertility in females. Endocrinology, 149(1), 20–27. 17947360 10.1210/en.2007-1084 PMC2194602

[tjp15279-bib-0037] Gong, D. W. , He, Y. , Karas, M. , & Reitman, M. (1997). Uncoupling protein‐3 is a mediator of thermogenesis regulated by thyroid hormone, beta3‐adrenergic agonists, and leptin. Journal of Biological Chemistry, 272(39), 24129–24132. 9305858 10.1074/jbc.272.39.24129

[tjp15279-bib-0038] Gorzek, J. F. , Hendrickson, K. C. , Forstner, J. P. , Rixen, J. L. , Moran, A. L. , & Lowe, D. A. (2007). Estradiol and tamoxifen reverse ovariectomy‐induced physical inactivity in mice. Medicine and Science in Sports and Exercise, 39(2), 248–256. 17277588 10.1249/01.mss.0000241649.15006.b8

[tjp15279-bib-0039] Gottsch, M. L. , Cunningham, M. J. , Smith, J. T. , Popa, S. M. , Acohido, B. V. , Crowley, W. F. , Seminara, S. , Clifton, D. K. , & Steiner, R. A. (2004). A role for kisspeptins in the regulation of gonadotropin secretion in the mouse. Endocrinology, 145(9), 4073–4077. 15217982 10.1210/en.2004-0431

[tjp15279-bib-0040] Greendale, G. A. , Han, W. , Finkelstein, J. S. , Burnett‐Bowie, S. M. , Huang, M. , Martin, D. , & Karlamangla, A. S. (2021). Changes in regional fat distribution and anthropometric measures across the menopause transition. Journal of Clinical Endocrinology and Metabolism, 106(9), 2520–2534. 34061966 10.1210/clinem/dgab389 PMC8372653

[tjp15279-bib-0041] Grefhorst, A. , van den Beukel, J. C. , van Houten, E. L. , Steenbergen, J. , Visser, J. A. , & Themmen, A. P. (2015). Estrogens increase expression of bone morphogenetic protein 8b in brown adipose tissue of mice. Biology of Sex Differences, 6(1), 7. 25866617 10.1186/s13293-015-0025-y PMC4392498

[tjp15279-bib-0042] Hazell, G. G. , Yao, S. T. , Roper, J. A. , Prossnitz, E. R. , O'Carroll, A. M. , & Lolait, S. J. (2009). Localisation of GPR30, a novel G protein‐coupled oestrogen receptor, suggests multiple functions in rodent brain and peripheral tissues. Journal of Endocrinology, 202(2), 223–236. 19420011 10.1677/JOE-09-0066 PMC2710976

[tjp15279-bib-0043] Heine, P. A. , Taylor, J. A. , Iwamoto, G. A. , Lubahn, D. B. , & Cooke, P. S. (2000). Increased adipose tissue in male and female estrogen receptor‐alpha knockout mice. Proceedings of the National Academy of Sciences, USA, 97(23), 12729–12734. 10.1073/pnas.97.23.12729 PMC18832 11070086

[tjp15279-bib-0044] Henry, B. A. , Andrews, Z. B. , Rao, A. , & Clarke, I. J. (2011). Central leptin activates mitochondrial function and increases heat production in skeletal muscle. Endocrinology, 152(7), 2609–2618. 21558317 10.1210/en.2011-0143

[tjp15279-bib-0045] Henry, B. A. , Dunshea, F. R. , Gould, M. , & Clarke, I. J. (2008). Profiling postprandial thermogenesis in muscle and fat of sheep and the central effect of leptin administration. Endocrinology, 149(4), 2019–2026. 18162524 10.1210/en.2007-1311

[tjp15279-bib-0046] Heymsfield, S. B. , Greenberg, A. S. , Fujioka, K. , Dixon, R. M. , Kushner, R. , Hunt, T. , Lubina, J. A. , Patane, J. , Self, B. , Hunt, P. , & McCamish, M. (1999). Recombinant leptin for weight loss in obese and lean adults: A randomized, controlled, dose‐escalation trial. JAMA, 282(16), 1568–1575. 10546697 10.1001/jama.282.16.1568

[tjp15279-bib-0047] Hilse, K. E. , Kalinovich, A. V. , Rupprecht, A. , Smorodchenko, A. , Zeitz, U. , Staniek, K. , Erben, R. G. , & Pohl, E. E. (2016). The expression of UCP3 directly correlates to UCP1 abundance in brown adipose tissue. Biochimica et Biophysica Acta, 1857(1), 72–78. 26518386 10.1016/j.bbabio.2015.10.011 PMC7115856

[tjp15279-bib-0048] Hirosawa, M. , Minata, M. , Harada, K. H. , Hitomi, T. , Krust, A. , & Koizumi, A. (2008). Ablation of estrogen receptor alpha (ERalpha) prevents upregulation of POMC by leptin and insulin. Biochemical and Biophysical Research Communications, 371(2), 320–323. 18439911 10.1016/j.bbrc.2008.04.073

[tjp15279-bib-0049] Hoene, M. , Li, J. , Haring, H. U. , Weigert, C. , Xu, G. , & Lehmann, R. (2014). The lipid profile of brown adipose tissue is sex‐specific in mice. Biochimica et Biophysica Acta, 1841(10), 1563–1570. 10.1016/j.bbalip.2014.08.003 25128765

[tjp15279-bib-0050] Hong, J. , Stubbins, R. E. , Smith, R. R. , Harvey, A. E. , & Nunez, N. P. (2009). Differential susceptibility to obesity between male, female and ovariectomized female mice. Nutrition Journal, 8(1), 11. 19220919 10.1186/1475-2891-8-11 PMC2650703

[tjp15279-bib-0051] Hubbard, K. , Shome, A. , Sun, B. , Pontre, B. , McGregor, A. , & Mountjoy, K. G. (2019). Chronic high‐fat diet exacerbates sexually dimorphic Pomctm1/tm1 mouse obesity. Endocrinology, 160(5), 1081–1096. 30997487 10.1210/en.2018-00924 PMC6469954

[tjp15279-bib-0052] Ikeda, K. , Maretich, P. , & Kajimura, S. (2018). The common and distinct features of brown and beige adipocytes. Trends in Endocrinology and Metabolism, 29(3), 191–200. 29366777 10.1016/j.tem.2018.01.001 PMC5826798

[tjp15279-bib-0053] Izumo, N. , Ishibashi, Y. , Ohba, M. , Morikawa, T. , & Manabe, T. (2012). Decreased voluntary activity and amygdala levels of serotonin and dopamine in ovariectomized rats. Behavioural Brain Research, 227(1), 1–6. 22056749 10.1016/j.bbr.2011.10.031

[tjp15279-bib-0054] Jones, M. E. , Thorburn, A. W. , Britt, K. L. , Hewitt, K. N. , Wreford, N. G. , Proietto, J. , Oz, O. K. , Leury, B. J. , Robertson, K. M. , Yao, S. , & Simpson, E. R. (2000). Aromatase‐deficient (ArKO) mice have a phenotype of increased adiposity. Proceedings of the National Academy of Sciences, USA, 97(23), 12735–12740. 10.1073/pnas.97.23.12735 PMC18833 11070087

[tjp15279-bib-0055] Justo, R. , Frontera, M. , Pujol, E. , Rodriguez‐Cuenca, S. , Llado, I. , Garcia‐Palmer, F. J. , Roca, P. , & Gianotti, M. (2005). Gender‐related differences in morphology and thermogenic capacity of brown adipose tissue mitochondrial subpopulations. Life Sciences, 76(10), 1147–1158. 15620578 10.1016/j.lfs.2004.08.019

[tjp15279-bib-0056] Karastergiou, K. , Smith, S. R. , Greenberg, A. S. , & Fried, S. K. (2012). Sex differences in human adipose tissues – The biology of pear shape. Biology of Sex Differences, 3(1), 13. 22651247 10.1186/2042-6410-3-13 PMC3411490

[tjp15279-bib-0057] Kemnitz, J. W. , Gibber, J. R. , Lindsay, K. A. , & Eisele, S. G. (1989). Effects of ovarian hormones on eating behaviors, body weight, and glucoregulation in rhesus monkeys. Hormones and Behavior, 23(2), 235–250. 2663699 10.1016/0018-506x(89)90064-0

[tjp15279-bib-0058] Khan, S. S. , Ning, H. , Wilkins, J. T. , Allen, N. , Carnethon, M. , Berry, J. D. , Sweis, R. N. , & Lloyd‐Jones, D. M. (2018). Association of body mass index with lifetime risk of cardiovascular disease and compression of morbidity. JAMA Cardiology, 3(4), 280–287. 29490333 10.1001/jamacardio.2018.0022 PMC5875319

[tjp15279-bib-0059] Kim, S. N. , Jung, Y. S. , Kwon, H. J. , Seong, J. K. , Granneman, J. G. , & Lee, Y. H. (2016). Sex differences in sympathetic innervation and browning of white adipose tissue of mice. Biology of Sex Differences, 7(1), 67. 27990249 10.1186/s13293-016-0121-7 PMC5148917

[tjp15279-bib-0060] Kirkby, J. , Metcalf, B. S. , Jeffery, A. N. , O'Riordan, C. F. , Perkins, J. , Voss, L. D. , & Wilkin, T. J. (2004). Sex differences in resting energy expenditure and their relation to insulin resistance in children (EarlyBird 13). American Journal of Clinical Nutrition, 80(2), 430–435. 15277166 10.1093/ajcn/80.2.430

[tjp15279-bib-0061] Koch, M. (1990). Effects of treatment with estradiol and parental experience on the number and distribution of estrogen‐binding neurons in the ovariectomized mouse brain. Neuroendocrinology, 51(5), 505–514. 2112728 10.1159/000125384

[tjp15279-bib-0062] Krause, W. C. , Rodriguez, R. , Gegenhuber, B. , Matharu, N. , Rodriguez, A. N. , Padilla‐Roger, A. M. , Toma, K. , Herber, C. B. , Correa, S. M. , Duan, X. , Ahituv, N. , Tollkuhn, J. , & Ingraham, H. A. (2021). Oestrogen engages brain MC4R signalling to drive physical activity in female mice. Nature, 599(7883), 131–135. 34646010 10.1038/s41586-021-04010-3 PMC9113400

[tjp15279-bib-0063] Lean, M. E. , James, W. P. , Jennings, G. , & Trayhurn, P. (1986). Brown adipose tissue uncoupling protein content in human infants, children and adults. Clinical Science, 71(3), 291–297. 3757433 10.1042/cs0710291

[tjp15279-bib-0064] Lidell, M. E. (2019). Brown adipose tissue in human infants. Handbook of Experimental Pharmacology, 251, 107–123. 29675580 10.1007/164_2018_118

[tjp15279-bib-0065] Lightfoot, J. T. , EJC, D. E. G. , Booth, F. W. , Bray, M. S. , M, D. , Kaprio, J. , Kelly, S. A. , Pomp, D. , Saul, M. C. , Thomis, M. A. , Garland, T., Jr. , & Bouchard, C. (2018). Biological/genetic regulation of physical activity level: Consensus from GenBioPAC. Medicine and Science in Sports and Exercise, 50(4), 863–873. 29166322 10.1249/MSS.0000000000001499 PMC6481631

[tjp15279-bib-0066] Lyons, P. M. , Truswell, A. S. , Mira, M. , Vizzard, J. , & Abraham, S. F. (1989). Reduction of food intake in the ovulatory phase of the menstrual cycle. American Journal of Clinical Nutrition, 49(6), 1164–1168. 2729155 10.1093/ajcn/49.6.1164

[tjp15279-bib-0067] Marraudino, M. , Carrillo, B. , Bonaldo, B. , Llorente, R. , Campioli, E. , Garate, I. , Pinos, H. , Garcia‐Segura, L. M. , Collado, P. , & Grassi, D. (2021). G Protein‐coupled estrogen receptor immunoreactivity in the rat hypothalamus is widely distributed in neurons, astrocytes, and oligodendrocytes, fluctuates during the estrous cycle, and is sexually dimorphic. Neuroendocrinology, 111(7), 660–677. 32570260 10.1159/000509583

[tjp15279-bib-0068] Martinez de Morentin, P. B. , Gonzalez‐Garcia, I. , Martins, L. , Lage, R. , Fernandez‐Mallo, D. , Martinez‐Sanchez, N. , Ruiz‐Pino, F. , Liu, J. , Morgan, D. A. , Pinilla, L. , Gallego, R. , Saha, A. K. , Kalsbeek, A. , Fliers, E. , Bisschop, P. H. , Dieguez, C. , Nogueiras, R. , Rahmouni, K. , Tena‐Sempere, M. , & Lopez, M. (2014). Estradiol regulates brown adipose tissue thermogenesis via hypothalamic AMPK. Cell Metabolism, 20(1), 41–53. 24856932 10.1016/j.cmet.2014.03.031 PMC4082097

[tjp15279-bib-0069] Melanson, E. L. , Gavin, K. M. , Shea, K. L. , Wolfe, P. , Wierman, M. E. , Schwartz, R. S. , & Kohrt, W. M. (2015). Regulation of energy expenditure by estradiol in premenopausal women. Journal of Applied Physiology, 119(9), 975–981. 26338457 10.1152/japplphysiol.00473.2015 PMC4628992

[tjp15279-bib-0070] Mokdad, A. H. , Ford, E. S. , Bowman, B. A. , Dietz, W. H. , Vinicor, F. , Bales, V. S. , & Marks, J. S. (2003). Prevalence of obesity, diabetes, and obesity‐related health risk factors, 2001. JAMA, 289(1), 76–79. 12503980 10.1001/jama.289.1.76

[tjp15279-bib-0071] Musatov, S. , Chen, W. , Pfaff, D. W. , Mobbs, C. V. , Yang, X. J. , Clegg, D. J. , Kaplitt, M. G. , & Ogawa, S. (2007). Silencing of estrogen receptor alpha in the ventromedial nucleus of hypothalamus leads to metabolic syndrome. Proceedings of the National Academy of Sciences, USA, 104(7), 2501–2506. 10.1073/pnas.0610787104 PMC1892990 17284595

[tjp15279-bib-0072] Olofsson, L. E. , Pierce, A. A. , & Xu, A. W. (2009). Functional requirement of AgRP and NPY neurons in ovarian cycle‐dependent regulation of food intake. Proceedings of the National Academy of Sciences, USA, 106(37), 15932–15937. 10.1073/pnas.0904747106 PMC2747221 19805233

[tjp15279-bib-0073] Ouellet, V. , Routhier‐Labadie, A. , Bellemare, W. , Lakhal‐Chaieb, L. , Turcotte, E. , Carpentier, A. C. , & Richard, D. (2011). Outdoor temperature, age, sex, body mass index, and diabetic status determine the prevalence, mass, and glucose‐uptake activity of ^18^F‐FDG‐detected BAT in humans. Journal of Clinical Endocrinology and Metabolism, 96(1), 192–199. 20943785 10.1210/jc.2010-0989

[tjp15279-bib-0074] Petrovic, N. , Walden, T. B. , Shabalina, I. G. , Timmons, J. A. , Cannon, B. , & Nedergaard, J. (2010). Chronic peroxisome proliferator‐activated receptor γ (PPARγ) activation of epididymally derived white adipocyte cultures reveals a population of thermogenically competent, UCP1‐containing adipocytes molecularly distinct from classic brown adipocytes. Journal of Biological Chemistry, 285, 7153–7164. 20028987 10.1074/jbc.M109.053942 PMC2844165

[tjp15279-bib-0075] Poehlman, E. T. (2002). Menopause, energy expenditure, and body composition. Acta Obstetricia et Gynecologica Scandinavica, 81(7), 603–611. 12190834 10.1034/j.1600-0412.2002.810705.x

[tjp15279-bib-0076] Pralong, F. P. , Gonzales, C. , Voirol, M. J. , Palmiter, R. D. , Brunner, H. R. , Gaillard, R. C. , Seydoux, J. , & Pedrazzini, T. (2002). The neuropeptide Y Y1 receptor regulates leptin‐mediated control of energy homeostasis and reproductive functions. FASEB Journal, 16(7), 712–714. 11978737 10.1096/fj.01-0754fje

[tjp15279-bib-0077] Pritchard, L. E. , Armstrong, D. , Davies, N. , Oliver, R. L. , Schmitz, C. A. , Brennand, J. C. , Wilkinson, G. F. , & White, A. (2004). Agouti‐related protein (83–132) is a competitive antagonist at the human melanocortin‐4 receptor: no evidence for differential interactions with pro‐opiomelanocortin‐derived ligands. Journal of Endocrinology, 180(1), 183–191. 14709157 10.1677/joe.0.1800183

[tjp15279-bib-0078] Proietto, J. (2017). Obesity and weight management at menopause. Australian Family Physician, 46, 368–370. 28609591

[tjp15279-bib-0079] Prossnitz, E. R. , Arterburn, J. B. , & Sklar, L. A. (2007). GPR30: A G protein‐coupled receptor for estrogen. Molecular and Cellular Endocrinology, 265–266, 138–142. 10.1016/j.mce.2006.12.010 PMC1847610 17222505

[tjp15279-bib-0080] Quarta, C. , Claret, M. , Zeltser, L. M. , Williams, K. W. , Yeo, G. S. H. , Tschop, M. H. , Diano, S. , Bruning, J. C. , & Cota, D. (2021). POMC neuronal heterogeneity in energy balance and beyond: An integrated view. Nature Metabolism, 3(3), 299–308. 10.1038/s42255-021-00345-3 PMC8085907 33633406

[tjp15279-bib-0081] Reilly, A. M. , Zhou, S. , Panigrahi, S. K. , Yan, S. , Conley, J. M. , Sheets, P. L. , Wardlaw, S. L. , & Ren, H. (2019). Gpr17 deficiency in POMC neurons ameliorates the metabolic derangements caused by long‐term high‐fat diet feeding. Nutrition & Diabetes, 9(1), 29. 31611548 10.1038/s41387-019-0096-7 PMC6791877

[tjp15279-bib-0082] Rodriguez‐Cuenca, S. , Pujol, E. , Justo, R. , Frontera, M. , Oliver, J. , Gianotti, M. , & Roca, P. (2002). Sex‐dependent thermogenesis, differences in mitochondrial morphology and function, and adrenergic response in brown adipose tissue. Journal of Biological Chemistry, 277(45), 42958–42963. 12215449 10.1074/jbc.M207229200

[tjp15279-bib-0083] Rodriguez, G. , Moreno, L. A. , Sarria, A. , Pineda, I. , Fleta, J. , Perez‐Gonzalez, J. M. , & Bueno, M. (2002). Determinants of resting energy expenditure in obese and non‐obese children and adolescents. Journal of Physiology and Biochemistry, 58(1), 9–15. 12222749 10.1007/BF03179833

[tjp15279-bib-0084] Roepke, T. A. , Bosch, M. A. , Rick, E. A. , Lee, B. , Wagner, E. J. , Seidlova‐Wuttke, D. , Wuttke, W. , Scanlan, T. S. , Ronnekleiv, O. K. , & Kelly, M. J. (2010). Contribution of a membrane estrogen receptor to the estrogenic regulation of body temperature and energy homeostasis. Endocrinology, 151(10), 4926–4937. 20685867 10.1210/en.2010-0573 PMC2946146

[tjp15279-bib-0085] Roesch, D. M. (2006). Effects of selective estrogen receptor agonists on food intake and body weight gain in rats. Physiology & Behavior, 87(1), 39–44. 16181647 10.1016/j.physbeh.2005.08.035

[tjp15279-bib-0086] Rogers, N. H. , Perfield, J. W., 2nd , Strissel, K. J. , Obin, M. S. , & Greenberg, A. S. (2009). Reduced energy expenditure and increased inflammation are early events in the development of ovariectomy‐induced obesity. Endocrinology, 150(5), 2161–2168. 19179442 10.1210/en.2008-1405 PMC2671894

[tjp15279-bib-0087] Rosenbaum, M. , Pietrobelli, A. , Vasselli, J. R. , Heymsfield, S. B. , & Leibel, R. L. (2001). Sexual dimorphism in circulating leptin concentrations is not accounted for by differences in adipose tissue distribution. International Journal of Obesity and Related Metabolic Disorders, 25(9), 1365–1371. 11571601 10.1038/sj.ijo.0801730

[tjp15279-bib-0088] Rossouw, J. E. , Anderson, G. L. , Prentice, R. L. , LaCroix, A. Z. , Kooperberg, C. , Stefanick, M. L. , Jackson, R. D. , Beresford, S. A. , Howard, B. V. , Johnson, K. C. , Kotchen, J. M. , & Ockene, J. & Writing Group for the Women's Health Initiative I (2002). Risks and benefits of estrogen plus progestin in healthy postmenopausal women: Principal results From the Women's Health Initiative randomized controlled trial. JAMA, 288, 321–333. 12117397 10.1001/jama.288.3.321

[tjp15279-bib-0089] Rothwell, N. J. , & Stock, M. J. (1983). Luxuskonsumption, diet‐induced thermogenesis and brown fat: The case in favour. Clinical Science, 64(1), 19–23. 6337007 10.1042/cs0640019

[tjp15279-bib-0090] Saad, M. F. , Damani, S. , Gingerich, R. L. , Riad‐Gabriel, M. G. , Khan, A. , Boyadjian, R. , Jinagouda, S. D. , el‐Tawil, K. , Rude, R. K. , & Kamdar, V. (1997). Sexual dimorphism in plasma leptin concentration. Journal of Clinical Endocrinology and Metabolism, 82, 579–584. 9024258 10.1210/jcem.82.2.3739

[tjp15279-bib-0091] Saito, K. , Cao, X. , He, Y. , & Xu, Y. (2015). Progress in the molecular understanding of central regulation of body weight by estrogens. Obesity, 23(5), 919–926. 25865677 10.1002/oby.21099 PMC4414873

[tjp15279-bib-0092] Saito, M. , Okamatsu‐Ogura, Y. , Matsushita, M. , Watanabe, K. , Yoneshiro, T. , Nio‐Kobayashi, J. , Iwanaga, T. , Miyagawa, M. , Kameya, T. , Nakada, K. , Kawai, Y. , & Tsujisaki, M. (2009). High incidence of metabolically active brown adipose tissue in healthy adult humans: Effects of cold exposure and adiposity. Diabetes, 58(7), 1526–1531. 19401428 10.2337/db09-0530 PMC2699872

[tjp15279-bib-0093] Salinero, A. E. , Anderson, B. M. , & Zuloaga, K. L. (2018). Sex differences in the metabolic effects of diet‐induced obesity vary by age of onset. International Journal of Obesity, 42(5), 1088–1091. 29463918 10.1038/s41366-018-0023-3

[tjp15279-bib-0094] Santollo, J. , Wiley, M. D. , & Eckel, L. A. (2007). Acute activation of ER alpha decreases food intake, meal size, and body weight in ovariectomized rats. American Journal of Physiology. Regulatory, Integrative and Comparative Physiology, 293(6), R2194‐R2201. 17942491 10.1152/ajpregu.00385.2007

[tjp15279-bib-0095] Sari, C. I. , Eikelis, N. , Head, G. A. , Schlaich, M. , Meikle, P. , Lambert, G. , & Lambert, E. (2019). Android fat deposition and its association with cardiovascular risk factors in overweight young males. Frontiers in Physiology, 10, 1162. 31620011 10.3389/fphys.2019.01162 PMC6759693

[tjp15279-bib-0096] Schorr, M. , Dichtel, L. E. , Gerweck, A. V. , Valera, R. D. , Torriani, M. , Miller, K. K. , & Bredella, M. A. (2018). Sex differences in body composition and association with cardiometabolic risk. Biology of Sex Differences, 9(1), 28. 29950175 10.1186/s13293-018-0189-3 PMC6022328

[tjp15279-bib-0097] Shaklai, S. , Grafi‐Cohen, M. , Sharon, O. , Shefer, G. , Somjen, D. , & Stern, N. (2019). Estradiol 17‐β induces pancreatic beta‐cell proliferation through distinct estrogen receptors in a glucose dependent manner. OBM Transplant, 3(3), 1.

[tjp15279-bib-0098] Sharma, G. , & Prossnitz, E. R. (2021). Targeting the G protein‐coupled estrogen receptor (GPER) in obesity and diabetes. Endocrine and Metabolic Science, 2, 100080. 35321004 10.1016/j.endmts.2021.100080 PMC8936744

[tjp15279-bib-0099] Simon, J. J. , Wetzel, A. , Sinno, M. H. , Skunde, M. , Bendszus, M. , Preissl, H. , Enck, P. , Herzog, W. , & Friederich, H. C. (2017). Integration of homeostatic signaling and food reward processing in the human brain. JCI Insight, 2(15), e92970. 28768906 10.1172/jci.insight.92970 PMC5543916

[tjp15279-bib-0100] Smith, J. T. , Cunningham, M. J. , Rissman, E. F. , Clifton, D. K. , & Steiner, R. A. (2005). Regulation of Kiss1 gene expression in the brain of the female mouse. Endocrinology, 146(9), 3686–3692. 15919741 10.1210/en.2005-0488

[tjp15279-bib-0101] Souba, W. W. (1997). Nutritional support. New England Journal of Medicine, 336(1), 41–48. 8970939 10.1056/NEJM199701023360107

[tjp15279-bib-0102] Stengel, A. , & Tache, Y. (2012). Ghrelin – A pleiotropic hormone secreted from endocrine x/a‐like cells of the stomach. Frontiers in Neuroscience, 6, 24. 22355282 10.3389/fnins.2012.00024 PMC3280431

[tjp15279-bib-0103] Stincic, T. L. , Grachev, P. , Bosch, M. A. , Ronnekleiv, O. K. , & Kelly, M. J. (2018). Estradiol drives the anorexigenic activity of proopiomelanocortin neurons in female mice. eNeuro, 5(4), ENEURO.0103–18.2018. 30310864 10.1523/ENEURO.0103-18.2018 PMC6179576

[tjp15279-bib-0104] Todd, J. , Lawrenson, R. , Farmer, R. D. , Williams, T. J. , & Leydon, G. M. (1999). Venous thromboembolic disease and combined oral contraceptives: A re‐analysis of the MediPlus database. Human Reproduction, 14(6), 1500–1505. 10357966 10.1093/humrep/14.6.1500

[tjp15279-bib-0105] Tolson, K. P. , Garcia, C. , Delgado, I. , Marooki, N. , & Kauffman, A. S. (2016). Metabolism and energy expenditure, but not feeding or glucose tolerance, are impaired in young Kiss1r KO female mice. Endocrinology, 157(11), 4192–4199. 27649089 10.1210/en.2016-1501 PMC5086529

[tjp15279-bib-0106] Tolson, K. P. , Garcia, C. , Yen, S. , Simonds, S. , Stefanidis, A. , Lawrence, A. , Smith, J. T. , & Kauffman, A. S. (2014). Impaired kisspeptin signaling decreases metabolism and promotes glucose intolerance and obesity. Journal of Clinical Investigation, 124(7), 3075–3079. 24937427 10.1172/JCI71075 PMC4071390

[tjp15279-bib-0107] Tolson, K. P. , Marooki, N. , De Bond, J. P. , Walenta, E. , Stephens, S. B. Z. , Liaw, R. B. , Savur, R. , Wolfe, A. , Oh, D. Y. , Smith, J. T. , & Kauffman, A. S. (2020). Conditional knockout of kisspeptin signaling in brown adipose tissue increases metabolic rate and body temperature and lowers body weight. FASEB Journal, 34(1), 107–121. 31914628 10.1096/fj.201901600R PMC7202476

[tjp15279-bib-0108] Trouillet, A. C. , Ducroq, S. , Naule, L. , Capela, D. , Parmentier, C. , Radovick, S. , Hardin‐Pouzet, H. , & Mhaouty‐Kodja, S. (2022). Deletion of neural estrogen receptor alpha induces sex differential effects on reproductive behavior in mice. Communications Biology, 5(1), 383. 35444217 10.1038/s42003-022-03324-w PMC9021208

[tjp15279-bib-0109] Valle, A. , Garcia‐Palmer, F. J. , Oliver, J. , & Roca, P. (2007). Sex differences in brown adipose tissue thermogenic features during caloric restriction. Cellular Physiology and Biochemistry, 19(1–4), 195–204. 17310113 10.1159/000099207

[tjp15279-bib-0110] Van Pelt, R. E. , Jones, P. P. , Davy, K. P. , Desouza, C. A. , Tanaka, H. , Davy, B. M. , & Seals, D. R. (1997). Regular exercise and the age‐related decline in resting metabolic rate in women. Journal of Clinical Endocrinology and Metabolism, 82, 3208–3212. 9329340 10.1210/jcem.82.10.4268

[tjp15279-bib-0111] van Veen, J. E. , Kammel, L. G. , Bunda, P. C. , Shum, M. , Reid, M. S. , Massa, M. G. , Arneson, D. V. , Park, J. W. , Zhang, Z. , Joseph, A. M. , Hrncir, H. , Liesa, M. , Arnold, A. P. , Yang, X. , & Correa, S. M. (2020). Hypothalamic oestrogen receptor alpha establishes a sexually dimorphic regulatory node of energy expenditure. Nature Metabolism, 2(4), 351–363. 10.1038/s42255-020-0189-6 PMC7202561 32377634

[tjp15279-bib-0112] Velasco, I. , Leon, S. , Barroso, A. , Ruiz‐Pino, F. , Heras, V. , Torres, E. , Leon, M. , Ruohonen, S. T. , Garcia‐Galiano, D. , Romero‐Ruiz, A. , Sanchez‐Garrido, M. A. , Olhsson, C. , Castellano, J. M. , Roa, J. , Poutanen, M. , Pinilla, L. , Vazquez, M. J. , & Tena‐Sempere, M. (2019). Gonadal hormone‐dependent vs. ‐independent effects of kisspeptin signaling in the control of body weight and metabolic homeostasis. Metabolism, 98, 84–94. 31226351 10.1016/j.metabol.2019.06.007

[tjp15279-bib-0113] Walden, T. B. , Hansen, I. R. , Timmons, J. A. , Cannon, B. , & Nedergaard, J. (2012). Recruited vs. nonrecruited molecular signatures of brown, “brite,” and white adipose tissues. American Journal of Physiology Endocrinology and Metabolism, 302(1), E19–E31. 21828341 10.1152/ajpendo.00249.2011

[tjp15279-bib-0114] Wallen, W. J. , Belanger, M. P. , & Wittnich, C. (2001). Sex hormones and the selective estrogen receptor modulator tamoxifen modulate weekly body weights and food intakes in adolescent and adult rats. Journal of Nutrition, 131(9), 2351–2357. 11533278 10.1093/jn/131.9.2351

[tjp15279-bib-0115] Wang, A. , Luo, J. , Moore, W. , Alkhalidy, H. , Wu, L. , Zhang, J. , Zhen, W. , Wang, Y. , Clegg, D. J. , Bin, X. , Cheng, Z. , McMillan, R. P. , Hulver, M. W. , & Liu, D. (2016). GPR30 regulates diet‐induced adiposity in female mice and adipogenesis in vitro. Scientific Reports, 6(1), 34302. 27698362 10.1038/srep34302 PMC5048424

[tjp15279-bib-0116] Wang, C. , He, Y. , Xu, P. , Yang, Y. , Saito, K. , Xia, Y. , Yan, X. , Hinton, A., Jr. , Yan, C. , Ding, H. , Yu, L. , Shu, G. , Gupta, R. , Wu, Q. , Tong, Q. , Lagor, W. R. , Flores, E. R. , & Xu, Y. (2018). TAp63 contributes to sexual dimorphism in POMC neuron functions and energy homeostasis. Nature Communications, 9(1), 1544. 10.1038/s41467-018-03796-7 PMC5906443 29670083

[tjp15279-bib-0117] Wang, C. , & Xu, Y. (2019). Mechanisms for sex differences in energy homeostasis. Journal of Molecular Endocrinology, 62(2), R129–R143. 31130779 10.1530/JME-18-0165 PMC6528488

[tjp15279-bib-0118] Willesen, M. G. , Kristensen, P. , & Romer, J. (1999). Co‐localization of growth hormone secretagogue receptor and NPY mRNA in the arcuate nucleus of the rat. Neuroendocrinology, 70(5), 306–316. 10567856 10.1159/000054491

[tjp15279-bib-0119] Xu, Y. , Nedungadi, T. P. , Zhu, L. , Sobhani, N. , Irani, B. G. , Davis, K. E. , Zhang, X. , Zou, F. , Gent, L. M. , Hahner, L. D. , Khan, S. A. , Elias, C. F. , Elmquist, J. K. , & Clegg, D. J. (2011). Distinct hypothalamic neurons mediate estrogenic effects on energy homeostasis and reproduction. Cell Metabolism, 14(4), 453–465. 21982706 10.1016/j.cmet.2011.08.009 PMC3235745

[tjp15279-bib-0120] Zingaretti, M. C. , Crosta, F. , Vitali, A. , Guerrieri, M. , Frontini, A. , Cannon, B. , Nedergaard, J. , & Cinti, S. (2009). The presence of UCP1 demonstrates that metabolically active adipose tissue in the neck of adult humans truly represents brown adipose tissue. FASEB Journal, 23(9), 3113–3120. 19417078 10.1096/fj.09-133546

